# Recognizing Alzheimer's disease from perspective of oligodendrocytes: Phenomena or pathogenesis?

**DOI:** 10.1111/cns.14688

**Published:** 2024-03-22

**Authors:** Jingji Wang, Yilan Zhen, Jun Yang, Shaojie Yang, Guoqi Zhu

**Affiliations:** ^1^ Center for Xin'an Medicine and Modernization of Traditional Chinese Medicine of IHM, and Key Laboratory of Molecular Biology (Brain Diseases) Anhui University of Chinese Medicine Hefei China; ^2^ Acupuncture and Moxibustion Clinical Medical Research Center of Anhui Province The Second Affiliation Hospital of Anhui University of Chinese Medicine Hefei China; ^3^ The First Affiliation Hospital of Anhui University of Chinese Medicine Hefei China

**Keywords:** Alzheimer's disease, amyloid deposition, myelination, oligodendrocyte

## Abstract

**Background:**

Accumulation of amyloid beta, tau hyperphosphorylation, and microglia activation are the three highly acknowledged pathological factors of Alzheimer's disease (AD). However, oligodendrocytes (OLs) were also widely investigated in the pathogenesis and treatment for AD.

**Aims:**

We aimed to update the regulatory targets of the differentiation and maturation of OLs, and emphasized the key role of OLs in the occurrence and treatment of AD.

**Methods:**

This review first concluded the targets of OL differentiation and maturation with AD pathogenesis, and then advanced the key role of OLs in the pathogenesis of AD based on both clinic and basic experiments. Later, we extensively discussed the possible application of the current progress in the diagnosis and treatment of this complex disease.

**Results:**

Molecules involving in OLs’ differentiation or maturation, including various transcriptional factors, cholesterol homeostasis regulators, and microRNAs could also participate in the pathogenesis of AD. Clinical data point towards the impairment of OLs in AD patients. Basic research further supports the central role of OLs in the regulation of AD pathologies. Additionally, classic drugs, including donepezil, edaravone, fluoxetine, and clemastine demonstrate their potential in remedying OL impairment in AD models, and new therapeutics from the perspective of OLs is constantly being developed.

**Conclusions:**

We believe that OL dysfunction is one important pathogenesis of AD. Factors regulating OLs might be biomarkers for early diagnosis and agents stimulating OLs warrant the development of anti‐AD drugs.

## Graphical abstract

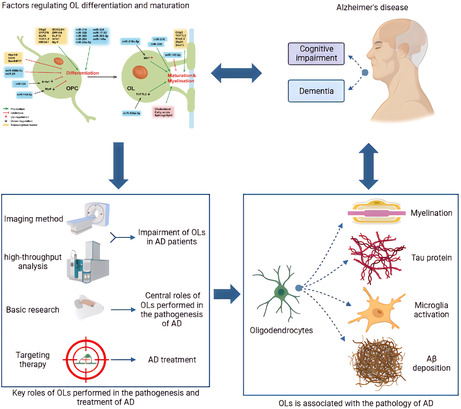

## 1 INTRODUCTION

Cognitive impairment or dementia is the main functional lesion of Alzheimer's disease (AD), which seriously affects the life quality of patients. In recent years, the incidence rate of AD has continued to increase. Approximately 6.7 million Americans (≥65 years) are living with Alzheimer's dementia today and this number is estimated to increase to 13.8 million by 2060.^1^ However, effective therapeutic drugs or intervention strategies are still lacking because the pathogenesis of AD has not yet been fully clarified.^2^ The inducements of AD include mutations in AD‐related genes such as *APP*, *PS1*, *tau*, *TARDBP*, and *apoE*, which accelerate the occurrence of dementia under the stimulation of specific external inducements.^3^, ^4^, ^5^ Non‐genetic environment factors including age, sex, lifestyle, occupational exposures to toxic substances, and cardiovascular‐ and cerebrovascular‐related diseases also contribute to the development and progression of AD.^6^, ^7^

The primary function of oligodendrocytes (OLs) in the CNS is to form myelin sheaths. After proliferation, oligodendrocyte precursor cells (OPCs) migrate to the designated area in the physiological condition or to the lesioned area in the pathological condition and differentiate into OLs, which play key roles in the process of myelination and remyelination, respectively.^8^ In addition to forming the myelin sheath, OLs also participate in several myelin‐independent aspects of development, function, and maintenance.^9^, ^10^ Munyeshyaka et al. reported that OLs and their progenitors were involved in different stages of memory, such as memory consolidation and recall in associative learning.^11^ In AD, OLs also precede to pathological factors such as amyloid deposition, tau hyperphosphorylation, and microglia activation.^12^ In the situation where a traditional idea cannot break through the treatment of AD, perhaps understanding AD from OLs would provide new insight into the diagnosis and treatment.

Classical neuropathological changes in AD, including the accumulation of amyloid plaque and the presence of neurofibrillary tangles, are responsible for neuronal loss and synaptic dysfunction,^13^, ^14^, ^15^, ^16^ but they may also induce death of OLs and myelin damage.^17^, ^18^ There is also evidence to show that myelin pathology may precede Aβ and tau pathologies in AD.^19^ Innovatively, myelin dysfunction even drives amyloid‐β deposition in AD models.^12^ A systematic review^20^ was published in 2019 on the critical roles of OLs in the development of AD. Considering the important relationship between OLs and AD, this review first concluded the targets of OL differentiation and maturation with AD pathogenesis and then advanced the key role of OLs in the pathogenesis of AD based on both clinical and basic experiments. Later, we extensively discussed the possible application of the current progress in the diagnosis and treatment of this complex disease.

## 2 FACTORS REGULATING OL DIFFERENTIATION AND MATURATION ARE ALTERED IN AD

**FIGURE 1:**
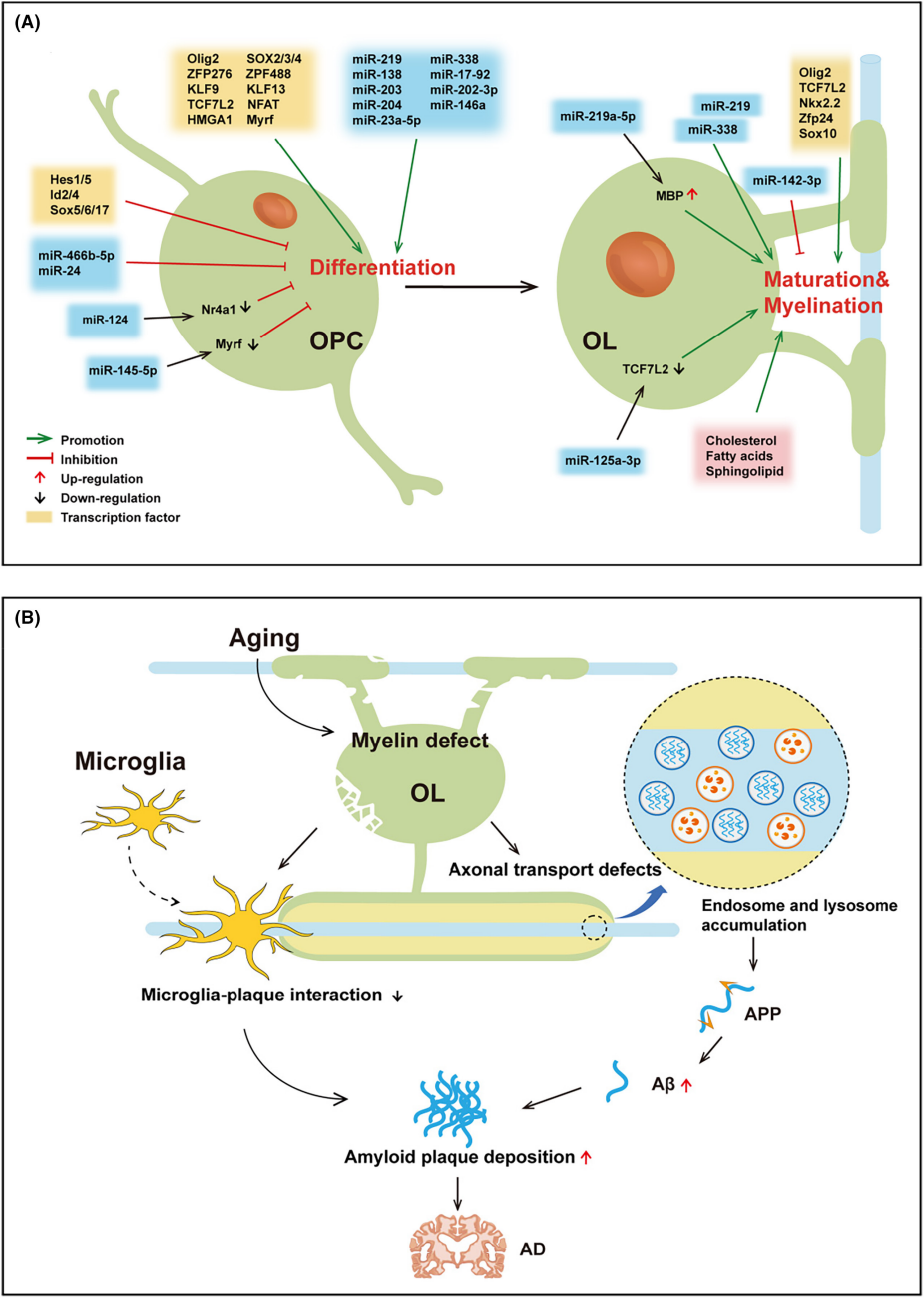
Factors regulating oligodendrocyte (OL) differentiation and maturation are potential therapeutic targets for Alzheimer's disease (AD). (A) Targets involved in OL differentiation and maturation, which are mainly divided into transcriptional factors, cholesterol homeostasis regulators, and microRNAs. (B) Potential effects of aging on OLs and amyloid‐β deposition.

Summarizing the literature from 2019 to the present, the targets involved in OL differentiation and maturation could be divided into three categories (Figure 1): transcriptional factors, cholesterol homeostasis regulators, and microRNAs (miRNAs). Interestingly, some molecules not only regulate the maturation and differentiation of OL but also participate in the pathological process of AD. Therefore, it is probable that those factors play significant roles in AD by regulating the maturation and differentiation of OLs and act as important targets for AD intervention. In addition, aging is the most critical factor causing AD and has a significant impact on the function of OLs. We will elaborate on the relevant regulatory roles in detail.

### 2.1 Role of transcriptional factors in AD

A large number of transcriptional factors (e.g., Olig2, NKX2.2, SOX2, KLF9, and NFAT) have been shown to promote OL differentiation, while many other transcriptional regulators (e.g., Hes1, Hes5, and Id2) inhibit OL differentiation.^21^, ^22^ Several studies have reported that those transcriptional factors are linked with the development of AD. According to the assay for transposase‐accessible chromatin with sequencing (ATAC‐Seq) data, olig2 and transcription factor 4 (TCF4), are highly expressed in the AD‐associated accessible chromatin regions in the hippocampus of the APPswe/PS1deltaE9 (APP/PS1) mice model.^23^ In addition, a recent study showed that Shenzhiling oral liquid‐containing serum promotes the differentiation and maturation of OLs in Aβ42‐induced OLN‐93 cells by increasing the expression of Olig2 and NKX2.2, indicating a critical role of Olig2 and NKX2.2 in AD.^24^ Transplantation of dimethyloxalylglycine‐preconditioned stem cells improved memory deficiency by increasing the levels of SOX2, nestin, and NeuroD in the hippocampus.^25^ Network analysis identified krupel like factor 9 (KLF9), Sp1 transcription factor (SP1) and chromodomain helicase DNA binding protein 1 (CHD1) as a key transcriptional regulator of a switch gene in the brain of AD patients.^26^ Although the role of nuclear factor of activated T‐cells (NFAT) in AD is unclear, the deletion of soluble epoxide hydrolase delays the progression of AD, partly by increasing the activity of nuclear factor kappa‐B (NF‐κB) and NFAT.^27^ A transcript analysis of the human entorhinal cortex from AD patients revealed increased expression of Hes1 in the disease progression, while Hes5 showed an up‐regulation in the intermediate stage of the disease and drop in severe AD.^28^ Furthermore, agomelatine, which is a promising candidate for the treatment of AD, could improve cognitive impairment by inhibiting Hes1/Notch1 signaling in mice.^29^ Low levels of Id2 were detected in the 5 × FAD mouse model of AD and human cadavers with AD. Id2 upregulation could augment microtubule stabilization through regulating acetylation of α‐tubulin lysine 40 and reconstitute axon growth, suggesting a possible beneficial role of Id2 in AD.^30^ Although the listed transcription factors, including those that promote transcription and those that inhibit transcription, can participate in the occurrence of AD, more research is needed to determine whether they participate in AD by regulating OLs, which means the causal relationship still needs to be further investigated.

### 2.2 Cholesterol homeostasis and ApoE in AD

The maturation of OLs into myelinating cells involves a series of morphological and functional changes and is characterized by the expression of myelin‐related proteins, such as myelin basic protein (MBP) and lipids. Cholesterol is one of the most studied lipids, as it is an essential building block for efficient myelin growth. There are many lines of evidence revealing a link between cholesterol homeostasis and AD pathology. A recent study showed that cholesterol esterification was impeded both in the plasma and cerebral spinal fluid (CSF) of AD patients; furthermore, the plasma cholesterol esterification biomarkers are closely related to AD biomarkers such as CSF Aβ1‐42.^31^

Apolipoprotein E (ApoE) ε4 is known to be the main risk factor for AD. Piccarducci et al. generated an in vitro ApoE ε4 cholinergic neuron model that recapitulates AD‐like features.^32^ Using this model, they demonstrated that ApoE ε4 induced neurotoxic effects on cholinergic neurons through neuronal cholesterol accumulation, acetylcholine dyshomeostasis, and diminished protein kinase C ε activation. Lee et al reported an ApoE ε4‐dependent lysosomal cholesterol accumulation, which impaired mitochondrial homeostasis and oxidative stress in human astrocytes.^33^ Neuronal ApoE ε4 may have roles in AD pathogenesis independent of cholesterol.^34^ The removal of neuronal ApoE diminished neurodegenerative disease‐associated subpopulations of neurons, oligodendrocytes, astrocytes and microglia.^35^ ApoE ε4 could also disrupt OLs differentiation by interfering with astrocyte‐derived lipid transport.^36^ In human APOE4 (hAPOE4) knock‐in mice, myelin lipid content is increased but the density of major myelin proteins, including MBP, MAG, and PLP is largely unchanged. They also found an unexpected but significant reduction of cell density of the OL lineage and an abnormal accumulation of OL precursors, suggesting a disruption of OL differentiation. Based on those data, the disruption of myelination in APOE4 carriers may represent a direct OL pathology, rather than an indirect consequence of amyloid plaque formation or neuronal loss.^37^

As reported by Blanchard et al,^38^ cholesterol is aberrantly deposited in oligodendrocytes and altered cholesterol localization in the ApoE brain coincides with reduced myelination. This study provided a single‐cell atlas describing the transcriptional effects of ApoE on the aging human brain and established a functional link between ApoE, cholesterol, myelination and memory in AD. The application of lipidated human ApoE protein was able to reduce the formation of myelinating OL in primary cell culture derived from *Apoe*‐knockout mice, suggesting that the disruption of myelination in *Apoe* carriers may represent a direct OL pathology.^37^ Moreover, altering the activity of phospholipase D3, a risk gene of late‐onset AD, significantly impacted APP processing and cholesterol metabolism.^39^

### 2.3 Involvement of miRNAs in AD

The regulation of OL development is highly influenced by miRNAs.^40^, ^41^ A set of miRNAs has been described to be positively regulated in the process of OL differentiation and maturation, including miR‐219, miR‐338, miR‐138, miR‐17‐92, miR‐202‐3p, miR‐203, miR‐204, miR‐146a, and miR‐23a‐5p. By contrast, miR‐145‐5p, miR‐466b‐5p, miR‐24, miR‐124, and miR‐142‐3p act as negative regulators of OL development.^42^, ^43^, ^44^ It should be pointed out that miR‐138 promotes the early phase of OL differentiation while delaying the later phase of OL maturation.^45^ Growing evidence suggests that some of these miRNAs may be important players in the development of AD by regulating Aβ accumulation (miR‐138,^46^, ^47^ miR‐146a^48^); tau hyperphosphorylation (miR‐138,^47^ miR219^49^); synaptic dysfunction (miR‐138^46^); neuroinflammation (miR‐146a^48^, ^50^); and dysfunctional autophagy (miR‐204^51^, ^52^). For example, miR‐138 overexpression enhanced Aβ production and tau phosphorylation in HT‐22 and SH‐SY5Y cells.^47^, ^53^ Consistently, Lu et al. further showed that miR‐318 not only modulated Aβ production by targeting Sirt1 but also induced synaptic and learning/memory deficits in APP/PS1 mice.^46^

In addition, many studies have reported that several miRNAs relating to OL development are dysregulated and may serve as biomarkers for developing diagnostic or therapeutic tools for AD, including miRNA‐146a,^54^, ^55^ miR124,^56^ and miR‐204^57^ (Table 1).

**TABLE 1:** microRNAs in Alzheimer's disease (AD) and effects on oligodendrocytes.

miRNAs	Changing trends in AD	Effects on oligodendrocyte
miR‐219	Inhibits tau phosphorylation by targeting TTBK1 and GSK‐3β^153^	Maturation,^154^ differentiation and myelination^155^
miR‐338	Alleviates neuronal apoptosis via directly targeting BCL2L11^156^, ^157^	Differentiation and maturation^158^
miR‐138	Promotes tau phosphorylation by targeting retinoic acid receptor alpha^159^	Differentiation^160^
miR‐17‐92	Abrogates autophagy‐mediated Amyloid‐β degradation^161^	Proliferation^162^
miR‐202‐3p	Reduced serum miR‐202 may promote the progression of Alzheimer's disease patients via targeting amyloid precursor protein^163^	Proliferation and differentiation^147^
miR‐204	Attenuated memory and synaptic deficits in APP/PS1 mice by targeting Nox4^164^	Proliferation and differentiation^165^
miR‐146a	Aggravates cognitive impairment and Alzheimer disease‐like pathology by triggering oxidative stress through MAPK signaling^166^	Differentiation^167^, ^168^
miR‐145‐5p	Regulates astrocyte‐neuron cross‐talk and protects against neuroinflammation^169^	Differentiation^167^
miR‐466b‐5p	Decreases in APPswe/PS δE9 double transgenic mice^170^	Differentiation^42^
miR‐24	Downregulation of miR‐24‐3p promoted cell proliferation and inhibited cell apoptosis^171^	Differentiation^43^
miR‐124	Acts as a target for Alzheimer's disease by regulating BACE1^172^	Proliferation^173^
miR‐142‐3p	Regulates cortical oligodendrocyte gene co‐expression networks associated with tauopathy^174^	Myelination^174^

### 2.4 Aging and the function of OLs

Depp et al. reported that abnormal myelin function drove amyloid‐β deposition.^12^ This viewpoint challenges the traditional belief that changes in OLs function and myelin sheath damage are only a concomitant phenomenon of the AD phenotype. Premature aging through transgenic methods can damage the myelin sheath, thereby promoting amyloid‐β deposition. In fact, the impact of aging on OLs has been widely recognized,^58^, ^59^ including inhibition of OLs proliferation and differentiation, cell death, etc (as reviewed in Ref. 60). Its mechanism may be related to promoting oxidative damage, inflammatory response and calcium overload. Interestingly, aging‐related impairment of OLs could be repaired by young CSF.^61^

A previous study demonstrated an essential role of IkappaB kinase (IKK)/NF‐κB signaling in mature, post‐mitotic OLs in senescence of these cells.^62^ Transmembrane protein 106B (TMEM106B), a regulator of lysosomal and oligodendrocyte function, was regulated with greatest effect size during aging. The increase in TMEM106B levels with aging was specific to carriers of the rs1990622‐A allele in the *TMEM106B* gene that increases risk for AD.^63^ Kaya et al reported that age‐related alterations of OLs cell state with a reduction in total oligodendrocyte density in aging murine white matter. Single‐cell RNA‐sequencing showed that interferon (IFN)‐responsive oligodendrocytes localized in proximity to CD8+ T cells in aging white matter. Further investigation demonstrated that CD8+ T‐cell‐induced IFN‐responsive oligodendrocytes are important modifiers of white matter aging.^64^

## 3 STUDY DESIGNS AND METHODS

We systematically searched for published literature (from 2019 to present) in PubMed and Web of Science by using oligodendrocyte and AD. Only studies published in English were considered.

**FIGURE 2:**
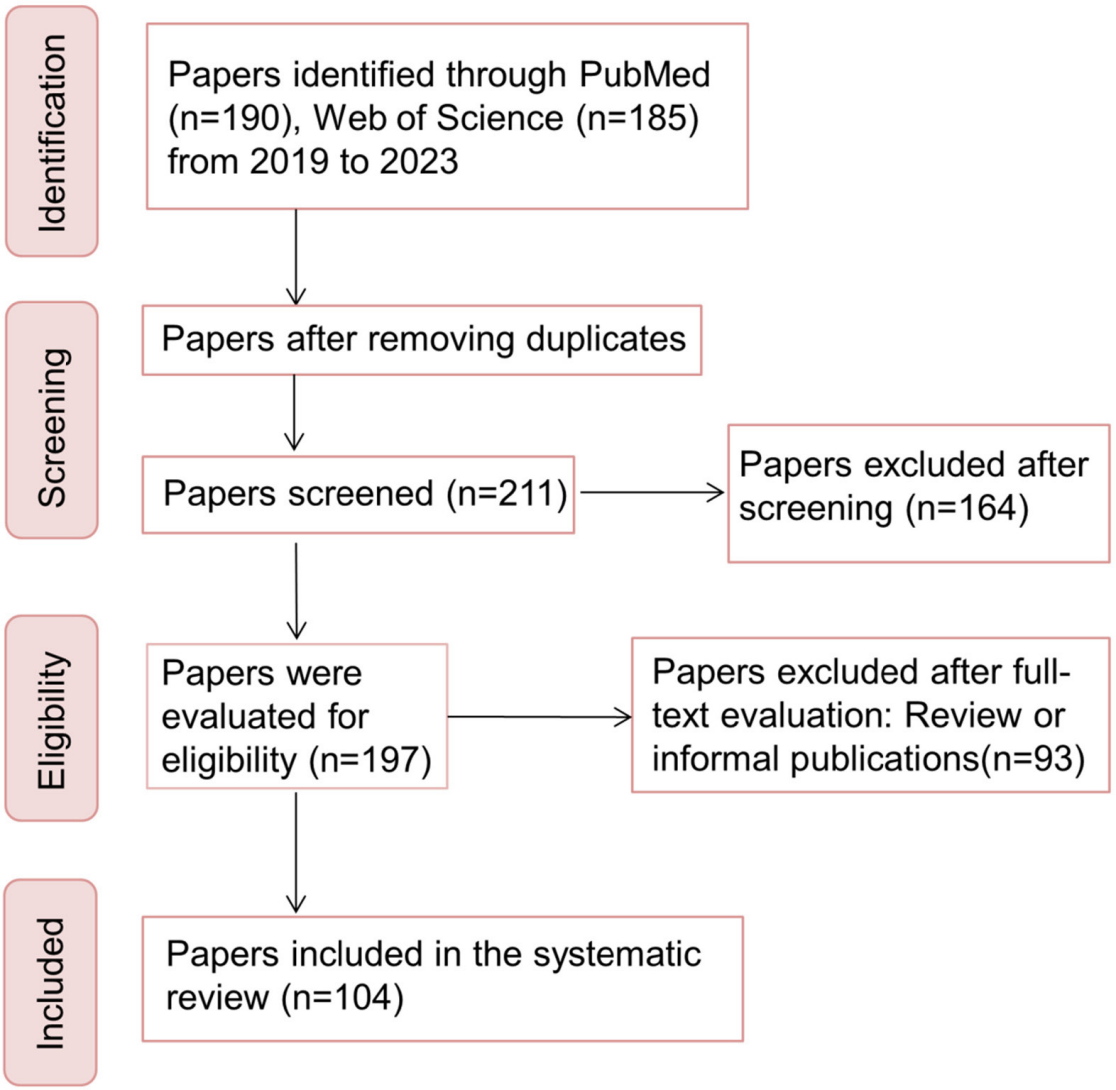
A schematic illustrates the study design. The literatures published from 2019 to present were searched in PubMed and Web of Science. A total of 211 studies were retrieved. Among them, 107 were excluded because they were either unrelated to this study or were informal published articles or review articles. Finally, 104 studies were included.

A total of 211 studies were retrieved. Among them, 107 were excluded because they were either unrelated to this study (*n* = 14) or were informal published articles or review articles (*n* = 93). Finally, 104 studies were included (Figure 2). We proposed OLs as the potential target in the pathogenesis and treatment of AD based on clinical and animal model investigations. Additionally, the mechanisms for the impairment and repair of OL were further discussed.

### 3.1 Medical imaging technology and high‐throughput analysis implicate the impairment of OLs in AD patients

**FIGURE 3:**
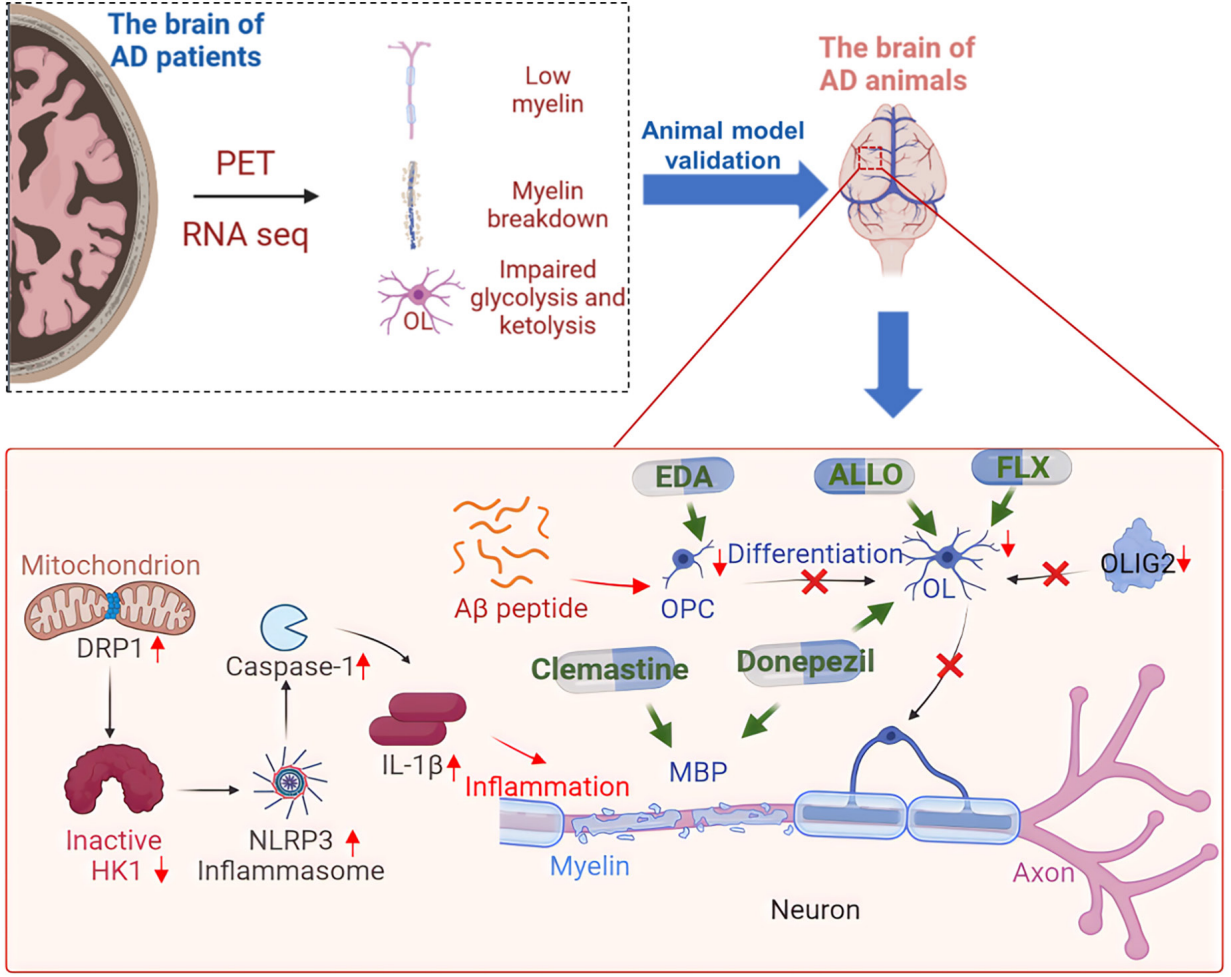
Oligodendrocytes (OLs) participate in the development and treatment of Alzheimer's disease (AD). Alterations of OLs were also observed in the blood sample or brain tissue of AD patients based on medical imaging technology and high‐throughput analysis. The Drp1‐HK1‐NLRP3 signaling axis is proven to be a key mechanism and therapeutic target for demyelination in AD.

Demyelination is one of the most important morphological alterations in both early and later stages of AD, and this point has been comprehensively discussed in previous studies.^65^ Current medical imaging methods or high‐throughput analysis of molecular markers further supports this point of view.^66^ For example, positron emission tomography (PET) technology provides the possibility to detect tau and Aβ in brain tissue and this method also indicates that high levels of myelin are associated with a low level of tau fibrils^67^ (Figure 3, Table 2). The demyelination level develops in the whole AD continuum, which indicates that this pathological process may be a related degenerative feature during disease progression.^68^

**TABLE 2:** Oligodendrocytes in demyelinating lesions in clinical Alzheimer's disease (AD) samples.

Sample source	Site of injury	OLs‐related mechanisms	References
Human brain	Gray matter	Myelin damage leads to the activation of tau‐targeting kinases entailing hyperphoshorylation of tau Impaired differentiation of OPCs Demyelination	[67]
Human brain	Temporal cortex, frontal pole, inferior frontal gyrus and parahippocampal gyrus	Impaired glycolytic and ketolytic pathways in OLs	[76]
Human brain	Cortex	The disorder of Drp1‐HK1‐NLRP3 signaling axis in mature OLs results in glycolytic deficiency, inflammation and demyelination	[77]
Human brain	White matter and deep gray matter	Higher iron content is associated with lower myelin content	[175]
Human brain	White matter and gray matter	Mutation of the APOE gene ε2 allele and ε4 allele corresponds to higher and lower myelin content in key brain regions respectively	[176]
Human brain	Prefrontal cortex	Altered cholesterol localization and homeostasis in ApoE oligodendrocytes may impair myelination	[38]

Alterations of OLs were also observed in the blood sample or brain tissue of AD patients. Using single‐nucleus RNA sequencing,^69^, ^70^, ^71^ transcriptomic and lipidomic profiles,^72^, ^73^ single‐cell transcriptomic analysis,^74^ and integrated proteomics,^75^ it was found that markers of OLs as well as gene expression in OLs was altered in AD patients. Interestingly, glycolytic and ketolytic pathways in OLs, but not in neurons, microglia, and astrocytes were significantly damaged in brain tissue of AD patients.^76^ Moreover, OL glycolytic stress could trigger the activation of inflammatory bodies and neuropathology of AD.^77^ Abnormality of OLs markers and myelin oligodendrocyte glycoprotein (MOG) was also found in human neural cell type‐specific extracellular vesicles.^78^, ^79^ Therefore, impairment of OLs could be measured in the blood of AD patients, and markers of OLs in extracellular vesicles might be useful to diagnose AD. Besides the changes of specific proteins in OLs, the DNA methylation of critical genes in OLs were also different in AD patients,^80^ implicating the epigenetic changes of OLs. These results provide important clinical evidence for diagnosis and treatment of AD from perspective of OLs.

### 3.2 The destruction of OPCs and OLs is a potential factor for AD

Loss of OLs leads to demyelination and this process is closely related to the impairment of learning and memory in AD animals^81^, ^82^ (Table 3). Chen et al reported that APP/PS1 mice in 8‐month old showed an increase in myelin drainage in the corpus callosum, cortex and hippocampus, indicating myelin loss. Reducing the mucosal M1 receptor in OPCs or systemic administration of the pro‐myelining drug in OL can repair learning and memory and hippocampal sharp wave ripples in AD mice, but did not alter Aβ clearance and deposition.^82^ This study indicates that OPCs‐dependent myelin renewal is sufficient to recover the memory impairment of APP/PS1 mice. Using the 5 × FAD mouse model, Angeli et al.^83^ demonstrated that the numbers of OPCs and mature OLs reduced in 5× FAD mouse model in 3‐ and 9‐months‐old. Dong et al.^84^ found that the expression of MBP was down‐regulated in the temporal lobe of APP/PS1 mice at 3 months of age compared with C57BL/6 mice, and this phenomenon became more obvious at 6 months, accompanied by other behavioral and pathological changes such as prolonged escape latency in the Morris water maze test, corpus callosum atrophy, increase of neuron‐glial antigen‐2 (NG2)‐immunoreactive cells, and decrease of monocarboxylic acid transporter (MCT1). To investigate the metabolic condition of AD brains, Chacon‐De‐La‐Rocha et al.^85^ evaluated the age‐related changes of OPCs in APP/PS1 mice and non‐transgenic age‐matched C57BL/6 mice. In this study, the authors also found that the numbers of OPCs and OPC sister cells decreased, but in 9‐month and 14‐month old APP/PS1 mice. In fact, the authors did not test the results using the younger mice. Chen et al.^86^ demonstrated that the expression of Olig2 decreased in the frontal cortex of 3xTg‐AD, accompanied by reduction of Olig2‐positive cells. Based on those data, early demyelination and OL dysfunction appear to precede the classical pathogenesis of AD, including accumulation of amyloid beta, tau hyperphosphorylation, and microglia activation. More important, OLs could be a target for the treatment of memory impairment.

**TABLE 3:** Oligodendrocytes in demyelinating lesions in AD animal models.

Animal model	Site of injury	Behavioral function change	Mechanism of damage	References
5× FAD mice (3, 6 and 9 months)	Corpus callosum, cortex and thalamus	Impaired hippocampus‐depended working memory and spatial learning	Disruption of astrocyte‐OL gap junctions could be a cause for the decrease of OPCs and mature Ols	[83]
APP/PS1 mice (3 and 6 months)	Corpus callosum and temporal lobe	Impaired spatial working memory	Reduction of MBP mRNA. Chaos and decrease of OLs	[84]
APP/PS1 mice (9 and 14 months)	Hippocampus	–	Decline of OPCs and myelination	[85]
3× Tg AD mice (3, 6 and 15 months)	Frontal cortex	–	Decrease of Olig2‐positive cells	[86]
5× FAD mice (3, 6 and 9 months)	Corpus callosum	Impaired short‐term cognitive ability	The disorder of Drp1‐HK1‐NLRP3 signaling axis in mature OLs results in glycolytic deficiency, inflammation and demyelination	[77]
3xTg AD mice (6 and 24 months)	Hippocampus	–	Decrease of OPCs and OPC sister cells	[87]
APP23 mice with chronic cerebral hypoperfusion (12 months)	Corpus callosum	–	Decrease of OLs and OPCs	[127]
APP/PS1 mice (8 months)	Hippocampus	Impaired hippocampus‐dependent spatial learning and memory abilities Impaired working and reference memory abilities	OL lineage senescence in the hippocampus	[128]
APP/PS1 mice (15 months)	Hippocampus and cortex	Impaired short‐term memory	Myelin degeneration Demyelination Decrease of OPCs and OLs	[129]
APP/PS1 mice (3 and 7.5 months)	Cerebellar tissue	Impaired short‐term spatial memory	OPCs senescence induced by Aβ	[130]
5× FAD mice (7, 10 and 15 months)	Brain tissue	–	Reactive oligodendrocyte response to Aβ pathology may be influenced by microgliosis	[71]

Zhang et al. found that the NLR family pyrin domain containing 3 (NLRP3)‐dependent gasdermin D‐associated inflammatory injury occurred in mature OLs of both AD patients and 5× FAD mice, concomitant with demyelination and axonal degeneration. The Drp1‐ hexokinase 1‐NLRP3 signaling axis is proven to be a key mechanism and therapeutic target for WM degeneration in AD.^77^ According to this study, Drp1 hyperactivation in mature OLs could induce a glycolytic defect by inhibiting HK1, triggering NLRP3‐associated inflammation (Figure 3). Vanzulli et al.^87^ analyzed the NG2 and MBP expression in the hippocampus of 3xTg‐AD mice at 6 and 24 months, and found that OPCs showed obvious morphological atrophy in 3xTg‐AD at 6 months, followed by morphological hypertrophy at 24 months. These results suggest that the destruction of OPCs and OLs is not only a potential factor for accelerated myelin loss and cognitive decline, but also an early pathological sign of AD, even preceding other AD pathologies. Interestingly, the degree of demyelinating lesions in the white matter correlated with the severity of symptoms of AD.^88^ The discovery of biomarkers provides an idea for disease diagnosis. Astrocyte markers and oligodendrocyte linear cell markers, such as *CCL2, YKL‐40, HGF, MIF, S100B, TSP2, LCN2*, and *serpinA3*, provide usable markers for early diagnosis of AD.^89^

### 3.3 Disruption of OLs contributes to early demyelination and damage of vasculature

Myelination, demyelination, and remyelination jointly maintain the balance and function of myelin. The mutation or variation of myelin formation‐related genes may lead to the development of abnormality of myelin sheath, such as that seen in Pelizaeus‐Merzbacher disease associated with an aberrant *PLP1* gene.^90^ In addition to the abnormal myelin formation caused by genetic factors, other environments or mutations of genes directly related to myelin can cause myelin lesion.^91^ Sufficient OPCs are required for the repair of demyelination in AD. With the aging process, the number of differentiated OPCs will be markedly reduced, affecting the process of remyelination.^58^, ^87^ The age of sporadic AD is >65 years, and the endogenous OPCs are insufficient for the treatment of AD. Of course, exogenous injection of OPCs may overcome this limitation.^92^ However, it is possible to regenerate OLs by adjusting the brain microenvironment, such as by the use of newborn animal cerebrospinal fluid.^61^ If sufficient OPCs are available, the differentiation and maturation regulation pathway of OLs is activated to guarantee myelin regeneration.^93^ Therefore, investigating the prevention and therapeutic strategies of AD from the perspective of OPC maturation and differentiation has important prospects.

Besides myelinating function, OLs can also interact with vascular cells to sustain the function of the blood–brain barrier (BBB).^94^ Aberrant OLs‐vascular cells interactions can damage BBB, which triggers CNS inflammation.^95^ Although the causes of AD and vascular dementia (VD) are not entirely same, they share common neurological abnormalities.^15^, ^16^ VD is mainly caused by abnormal vascular function, including inhibition of cerebral blood flow or destruction of the BBB. In fact, vascular abnormalities also induce the progression of AD.^96^, ^97^ Therefore, given the common mechanisms and intervention strategy, AD and VD are often considered simultaneously.^15^, ^98^ Additionally, OLs also participate in several myelin‐independent aspects of development, function, and maintenance.^10^, ^11^ Therefore, the function of non‐myelinating OLs should be further investigated in the pathogenesis and treatment of AD.

### 3.4 OLs also participate in other AD pathologies

**FIGURE 4:**
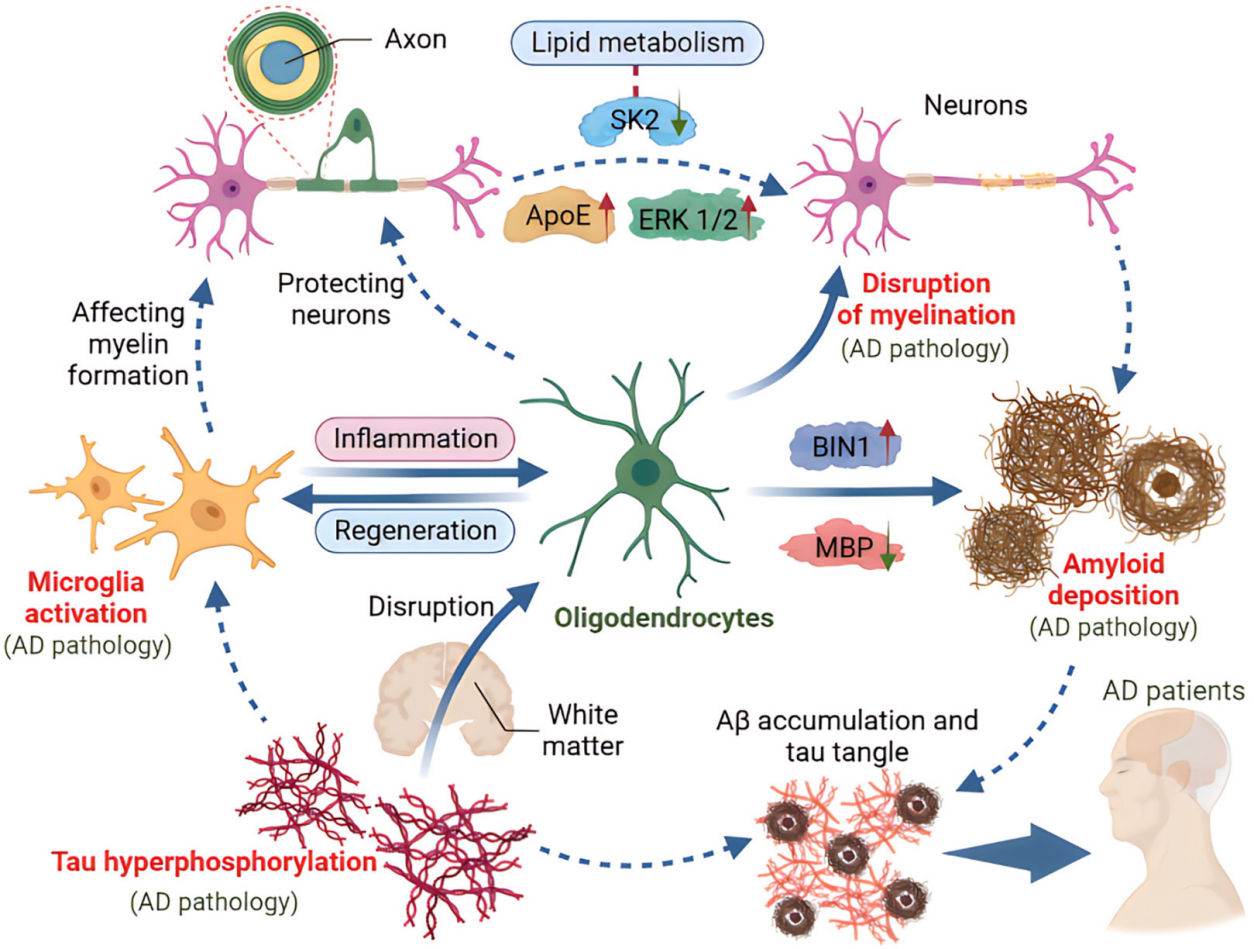
Oligodendrocytes (OLs) exhibit protection against Alzheimer's disease (AD) pathologies. In addition to protecting neurons by myelination, OLs also participate in other AD pathologies through regulating amyloid deposition, tau propagation and microglia activation.

In addition to forming myelination, OLs also participate in other AD pathologies (Figure 4). Bridging integrator 1 (BIN1) is considered the second most recent genetic risk factor in advanced AD. According to literatures, *BIN1* expresses an adapter protein that regulates dynamic changes in the membrane by directly binding to tau, affecting endocytosis and neurotransmitter vessel release.^62^, ^99^ BIN1 is expressed in various cells in the brain, including neurons and glial cells, and plays a variety of biological activities. In microglia, BIN1 regulates the inflammatory response of microglia.^100^ In neurons and astrocyte, BIN1 correlated with tau pathology.^101^, ^102^ In neurons, BIN1 can also regulate the release of presynaptic neurotransmitters, affecting memory consolidation.^103^ It can also affect postsynaptic trafficking, thereby regulating glutamate neuron signal transduction.^104^ On the contrary, conditional knockout of BIN1 in excitatory neurons does not affect Aβ generation.^105^ BIN1 is also expressed in mature Ols, and the aberrant accumulation of BIN1 in OLs was associated with extracellular Aβ deposition.^106^ In another study, investigators showed that MBP knockout mice exhibited increased Aβ oligomer production.^107^ As we mentioned earlier, aging can disrupt the structure and function of the myelin sheath, and myelin dysfunction causes the accumulation of the Aβ‐producing machinery within axonal swellings and increases the cleavage of cortical amyloid precursor protein. Because of this, age‐dependent myelin abnormality is thought to be a critical upstream AD risk factor.^12^

Altered lipid metabolism and Aβ accumulation are key pathogenic factors of AD. Lei et al. found that sphingosine kinase 2 (SK2) is a key regulator of endosomal lipid metabolism, and loss of SK2 activity could impair OLs by disturbing endosomal lipid metabolism.^108^ Tau abnormality is also another critical factor related to AD pathology. While most researchers focused on neuronal tau, Hirota et al. reported that AD‐related p‐tau181 signals localized to demyelinated axons of parvalbumin‐positive GABAergic interneurons in an *App* knock‐in mouse model of AD.^109^ Of note, oligodendroglial tau could propagate along WM tracts, resulting in the loss of OLs. Moreover, this process was independent of neuronal tau.^110^ Additionally, high levels of myelin are associated with low susceptibility of the accumulation of fibrillar tau,^67^ further supporting the potential causality between OLs and tau. In the research of tau pathology, Emerson et al. used R955‐hTau transgenic rates and found that the changes in myelin pathology preceded tau pathology.^111^ In addition to Aβ deposition and tau pathology, disruption of OLs also correlates with microglia activation.^112^ As reported, progranulin deficiency results in sex‐dependent alterations in microglia, which was followed by OLs disruption.^113^ In addition to their function in the form of myelination, disruption of OLs also correlates with other AD pathologies. For example, genes in OLs such as *BIN1* and *MBP* affect extracellular Aβ deposition.^12^, ^106^, ^107^ While it is well‐known that there are many theories about AD pathogenesis, the deposition of senile plaques plays a very important role, which is why scientists have since long focused more on Aβ to identify effective therapeutic agents.^114^, ^115^ Fortunately, aducanumab has been approved for clinical use in the treatment of AD. As an alternative treatment of AD, traditional Chinese medicine has also demonstrated the potential to treat or prevent AD.^4^, ^5^, ^14^, ^16^ If, as mentioned in the above literature, OLs can also regulate Aβ, treatment strategies can be designed around OLs to suppress Aβ deposition.

In addition to Aβ deposition, tau abnormality is also considered a critical factor in AD pathology. For example, post‐translational modification of tau, especially hyperphosphorylation, can affect neuronal synaptic plasticity and survival.^116^, ^117^ Besides, tau can also epigenetically remodel the neuron‐glial cross‐talk in AD.^118^ Therefore, neuronal tau is an important breakthrough in the treatment of AD. Immunotherapy targeting tau has demonstrated important potential in the treatment of AD.^119^, ^120^ Recent research suggests that OLs tau can propagate along WM tracts, resulting in the loss of OLs.^110^ OL tau may also be an important mechanism for the occurrence of AD, which enriches the tau hypothesis in AD.^121^, ^122^, ^123^

Disease‐associated oligodendrocytes (DAOs) have also attracted considerable attention. Pak et al.^124^ reported AD‐associated oligodendrocyte subtypes via single‐cell expression profiling analysis. The cellular changes of DAOs could provide useful therapeutic approaches to treat AD.^94^ For example, investigators suggested Erk1/2 as the critical signaling pathway regulating DAOs, and inhibition of Erk1/2 remedied the DAOs and rescued impaired axonal myelination.^124^

### 3.5 Targeting therapy of OLs might be useful in the treatment of AD

Classic drugs, such as donepezil, edaravone, fluoxetine (FLX), and clemastine, have the potential to remedy the impairment of OLs and ameliorate AD‐related behaviors (Figure 2). For example, donepezil could significantly upregulate MBP and GST‐π expression in the corpus callosum in a cuprine‐induced demyelination mouse model.^125^ Interestingly, donepezil could also stimulate the differentiation of NSCs into OLs,^126^ implicating a new mechanism for the treatment of AD. Edaravone could enhance the proliferation of OPCs and improve the chronic cerebral hypoperfusion‐induced corpus callosum WM pathology in APP23 mice.^127^ Remarkably, in addition to cognitive requirements, other drugs such as antidepressants have also been used for the treatment of AD, and the mechanism is related to OL. Chao et al.^128^ used FLX as therapeutic intervention in 8‐month‐old male APP/PS1 mice for 2 months, and found that FLX delayed the aging of OL lineage cells in the hippocampus of APP/PS1 mice and promoted OL maturation. Clemastine, as an H1‐antihistamine, could increase the density of OPCs, OLs, and myelin in 15‐month‐old APP/PS1 mice.^129^

New therapeutics from the perspective of OLs are constantly being developed. Zhang et al.^130^ reported that Aβ‐induced OPC senescence played an important role in neuroinflammation and cognitive deficits in AD, and senolytic treatment (oral gavage with dasatinib and quercetin) had a potential therapeutic benefit in this aspect. Chen et al. reported that allopregnanolone promoted OL differentiation and demonstrated its potential in the treatment of AD.^86^ Additionally, small molecules with the potential to activate OLs have also demonstrated their efficacy in AD treatment.^131^ 27‐hydroxycholesterol promotes OL maturation and has the potential to treat AD behaviors.^132^ *Gardenia jasminoides J. Ellis* extract alleviated WM damage in APP/PS1 mice by promoting the differentiation of OPCs via suppressing neuroinflammation.^133^

In addition to drug treatment, physical therapy and gene therapy by targeting OLs also demonstrate great therapeutic potential against AD. Low‐intensity pulsed ultrasound therapy could also remedy the impairment of OLs in the AD mice model.^134^ Genetic deletion of oligodendroglial muscarinic M1 receptor could improve the learning and memory of APP/PS1 mice.

## 4 FUTURE PERSPECTIVES

In this review, we updated the regulatory mechanism of OL differentiation and maturation, listed a series of targets, and provided important clues for understanding the pathogenesis and intervention of OLs in AD. We believe this review can provide important ideas for new intervention strategies.

What induces OL dysfunction or cell loss in AD? There are three possible answers for this question: First, inflammatory reactions chronically occur during the whole period of AD, and similar to other demyelinating diseases, abnormalities in the inflammatory microenvironment are an important reason for OL loss.^135^ Second, senile plaques and tau tangles are the main pathological features of AD, which can activate microglia and then phagocytose OLs. Finally, a recent study pointed out that *APOE*, a key gene of sporadic AD, could cause demyelinating lesions by regulating cholesterol homeostasis and transport in OLs.^38^ In addition, the newly discovered AD‐related protein, TDP‐43, may also be a potential factor regulating OL function or causing cell loss.^136^ Based on this, approaching AD from the perspective of OLs would enrich its understanding and provide more diagnostic markers.

Microglial activation is an important mechanism of AD. Microglia‐sensing neuronal activity and the surrounding microenvironment enables them to optimize myelination by influencing early oligodendrogenesis.^137^ Sherafat et al. also reported that adjacent microglia promoted OLs expansion through neuropilin‐1.^138^ Giera et al. found that microglial transglutaminase‐2 drives myelination and myelin repair via G‐protein coupled receptor‐56 signal in OPCs.^139^ OLs, microglia, and astrocytes are considered the most common glia in the brain. By analyzing brain tissues of a mouse model of AD at 8 and 13 months of age, researchers found that disease‐associated microglia (DAM) were in close contact with amyloid‐beta plaques; whereas, disease‐associated OPCs are enriched in the outer shells surrounding the plaque‐DAM complex.^140^

Many factors regulate the differentiation and maturation of OLs, which can be divided into three categories, including transcription factors, cholesterol homeostasis regulating factors, and miRNAs.^141^, ^142^ Transcription factors can directly regulate the differentiation of OPCs, while cholesterol homeostasis‐regulating factors play a crucial role in the maturation process of OLs. In addition, miRNAs, as important non‐coding RNAs for epigenetic regulation, play a regulatory role in the expression of transcription factors and metabolic regulatory factors. These factors play a crucial role in the differentiation and maturation of OLs. At the same time, the abnormalities of these factors themselves can mediate the occurrence of AD, and correcting these factors can have an interventional effect on AD. For example, research reports have pointed out that miRNAs play an inhibitory or promoting role in the differentiation and maturation of OLs.^40^, ^41^ miRNAs play a special regulatory function in epigenetics and have multiple biological functions. In fact, miRNAs play an important role in the development of AD by regulating the survival of neurons, synaptic plasticity, and inflammatory response.^143^, ^144^, ^145^ By contrast, we found that there are only a few research reports on the involvement of miRNAs in the differentiation and maturation of OLs in AD.^146^, ^147^ How miRNAs regulate factors of OLs differentiation and maturation in AD is worth further exploration. Single‐cell sequencing technology provides important research means for the functional research of specific cells, and the gene expression regulation of specific cells provides novel ideas for revealing whether these factors participate in the occurrence or intervention of AD by influencing OL.^74^

This review systematically analyzes the prospect and potential of OLs as targeted therapy for AD. OLs can not only improve and repair the demyelination seen in AD but also affect other pathological changes of AD. However, when considering OLs, we must take into account other pathogenesis factors of AD. Simply regulating OLs is insufficient to achieve the purpose of myelin regeneration. Myelination is a relatively complex process, which is controlled by the interaction of multiple cells in the local microenvironment. McNamara et al. believed that microglia regulated the growth and integrity of myelin in the CNS.^148^ Wang et al. pointed out that the function of TREM2‐dependent microglia was crucial to myelin formation and subsequent neuroprotection,^149^ and the main reason was that TREM2‐dependent microglia showed defects in the migration and phagocytosis of myelin fragments. Zhou et al. reported that the supplementation of conjugated linoleic acid in vivo and in vitro can simulate these effects, including reducing inflammation and restoring the regeneration property of microglia, finally making better recovery of demyelinating injury and improving spatial learning function.^150^ In addition to glia, which directly affects the regeneration of myelin sheath, other types of cells have a direct role in the differentiation and maturation of OLs. Interestingly, astrocyte endfoot formation regulated by semaphorins 3a and 6a controls the termination of OPC perivascular migration.^151^ Enrich‐Bengoa et al. used transcriptome data to interpret the interaction between microglia and OPCs in the process of demyelination. In the cuprizone model, there are 47 ligands in microglia, 43 in OPC receptors, and 115 in OPC target genes participating in the microglia‐OPC crosstalk.^152^ Although this study focuses on demyelinating lesions rather than AD, it suggests the importance of interactive study of OLs and other types of cells.

Donepezil, edaravone, FLX, and clemastine have the potential to remedy OLs. However, we still do not know whether these drugs improve AD by repairing OLs, or whether the repair of OLs is only an accompanying phenomenon after improving AD. In the future, more refined experimental designs may be needed to prove the relevant causal relationships. In addition to these classical therapeutic agents, more specific experiments for manipulating OL are still needed to reveal the optimum targeted therapy of OLs.

## 5 CONCLUSION

This review summarizes the regulatory targets of the differentiation and maturation of OLs, and emphasizes the key role of OLs in the occurrence and treatment of AD. We believe that whether it is OLs damage caused by AD pathology or AD pathology caused by OLs, sufficient attention should be paid to the prevention and treatment of OLs in AD.

## AUTHOR CONTRIBUTIONS

JJW and GQZ designed the review. JJW, YLZ, JY, SJY and GQZ completed the writing of this paper.

## FUNDING INFORMATION

This research was supported by the National Natural Science Foundation of China (82004481), Anhui Natural Science Foundation (grant Nos. 2208085MH282), Research Funds of Center for Xin'an Medicine and Modernization of Traditional Chinese Medicine of IHM (2023CXMMTCM013 and 2023CXMMTCM021), the Talent Support Project of Anhui Universities (grant No.gxyqZD2022053), Key Project of Anhui Natural Science Research (grant No. 2022AH050462), and the Open Fund of Anhui Acupuncture and Moxibustion Clinical Medical Research Center (grant No. 2021zjzx08).

## CONFLICT OF INTEREST STATEMENT

The authors declare that there are no conflicts of interest.

## Data Availability

Data sharing is not applicable to this article as no new data were created or analyzed in this study.
